# Sparstolonin B nano-formulation attenuates LPS-induced lung injury

**DOI:** 10.3389/fphar.2025.1532391

**Published:** 2025-04-08

**Authors:** Qinghe Meng, Xiaojing Wang, Dandan Guo, Gary Zhang, Changying Shi, Adam Novak, Xiguang Yang, Juntao Luo, Robert N. Cooney

**Affiliations:** ^1^ Department of Surgery, State University of New York (SUNY), Upstate Medical University, Syracuse, NY, United States; ^2^ Sepsis Interdisciplinary Research Center (SIRC), State University of New York (SUNY), Upstate Medical University, Syracuse, NY, United States; ^3^ Department of Pharmacology, State University of New York (SUNY), Upstate Medical University, Syracuse, NY, United States

**Keywords:** sparstolonin B (nSsnB), LPS, ALI/ARDS, TLR-4, NF-κB, inflammation, nanomedicine

## Abstract

**Introduction:**

Nanomedicines can improve drug delivery and efficacy while reducing side effects. Our study examines the impact of a nano-formulation of Sparstolonin B (nSsnB), a TLR-4 antagonist, on LPS-induced inflammation in RAW264.7 cells and lung injury in mice.

**Methods:**

RAW264.7 cells were treated with LPS (1 μg/mL) ± nSsnB (2–64 μg/mL) for 24 h. Cell viability was assessed, cytokine levels in media were measured, and cell lysates were used to quantify NF-κB activation. C57BL/6 mice were treated with prophylactic intratracheal (IT) nSsnB (0.625 mg/kg) ± IT LPS (2.5 mg/kg). Blood and BALF were collected for cytokine, protein and cytological analysis. Lung histology was scored to evaluate lung injury. The relative abundance of MyD88 and phosphorylated NF-κB were measured in lung and HLL mice were used to measure NF-κB activation *in vivo*.

**Results:**

nSsnB demonstrated reduced toxicity vs. free SsnB. nSsnB ameliorated the LPS-induced increase in TNF-α, IL-6 and NF-κB P65 phosphorylation in RAW264.7 cells. LPS-treated mice revealed histologic ALI, elevated BALF neutrophils/macrophages/total protein, and increased levels of TNF-α/IL-6 in both BALF and plasma. Prophylactic nSsnB attenuated all these parameters in the LPS/nSsnB group. The increased levels of MyD88 and P-NF-κB P65 in lung from LPS-treated mice were reduced in the LPS/nSsnB group and nSsnB attenuated the increase in NF-κB activation induced by IT LPS in HLL mice.

**Conclusion:**

nSsnB demonstrates less toxicity than free SsnB and attenuates the effects of LPS on inflammation in RAW264.7 cells. Prophylactic nSsnB attenuates LPS-induced ALI by reducing inflammation via MyD88/NF-κB signaling pathways. Collectively these findings support the therapeutic potential of nano-formulated nSsnB for ALI treatment.

## 1 Introduction

Acute respiratory distress syndrome (ARDS), a severe form of acute lung injury (ALI) (Arora et al., Meyer et al., 2021), is characterized by widespread inflammation and damage to the alveolar-capillary barrier, leading to severe hypoxemia and respiratory failure. Current treatments for ARDS include mechanical ventilation to maintain oxygenation and supportive care like fluid resuscitation/vasopressors to maintain blood pressure, nutrition support and renal replacement therapy in severe cases (Diamond et al., 2025). While supportive care can provide life support, it does not address the underlying causes, and thus, does not completely prevent the complications of ARDS. Additionally, treatments such as mechanical ventilation and fluid management carry risks of complications. Medications such as corticosteroids, antibiotics, nitric oxide (Jiang et al.), and nonsteroidal anti-inflammatory drugs have been used in the treatment of ALI/ARDS, but their efficacy varies (Brun-Buisson et al., 2011; Cepkova and Matthay, 2006; Afshari et al., 2011). There are currently no specific drugs on the market for treating ALI/ARDS, and the efficacy and safety of existing treatments requires further validation (Banavasi et al., 2021). Moreover, research on drugs specifically targeting ALI/ARDS is relatively limited (Zhang et al., 2024), necessitating more clinical trials to assess their efficacy. Conceptually, therapies to target exaggerated systemic inflammation in ARDS could help to reduce injury to the lungs and other organs.

Toll-like receptors (TLRs) are a group of transmembrane proteins crucial for recognizing pathogenic threats and initiating an innate immune response (Nie et al., 2018). Given the continuous exposure of the lungs to various infectious agents, antigens, and host-derived danger signals, stromal and myeloid cells in the lung express a variety of TLRs (Arora et al., 2019). These receptors detect both endogenously derived damage-associated molecular patterns (DAMPs) and pathogen-associated molecular patterns (PAMPs), activating TLR-associated signaling pathways essential for host defense (Nie et al., 2018). Consequently, TLRs play a critical role in activating host defense mechanisms during microbial infections as well as non-infectious pulmonary disorders like interstitial lung disease and asthma (Kovach and Standiford, 2011). In particular, TLRs are vital for immune host defense against bacterial, fungal, and viral pathogens in the lungs (Jiang et al., 2006; Jiang et al., 2005; Chassin et al., 2009; Kovach and Standiford, 2011; Arora et al., 2019). Specifically, TLR4 is implicated in inflammatory conditions, suggesting the potential for disease management through TLR-targeted immunotherapeutic approaches (Mukherjee et al., 2016). In the lung injury, TLR signaling also plays a key role in the pathogenesis of diseases such as ARDS, chronic obstructive pulmonary disease (COPD), and pneumonia. Overactivation of TLRs can lead to excessive inflammation, contributing to tissue damage and worsening disease outcomes (Medzhitov, 2001; Takeda and Akira, 2005). Studies have shown that TLR4 antagonists can reduce inflammation and protect against lung injury in models of sepsis and acute lung injury (Shirey et al., 2013; Mukherjee et al., 2016). TLR signaling is mediated through the adaptor proteins MyD88, which trigger downstream inflammatory responses. Recent studies have shown that targeting these adaptors can attenuate TLR-induced lung injury. Inhibition of MyD88 signaling has been shown to reduce inflammation and improve recovery in animal models of LPS-induced lung injury (Fan et al., 2023). Several studies have indicated that altering TLR signaling to dampen inflammation could serve as an effective strategy for mitigating ALI/ARDS (Huang et al., 2024).

Sparstolonin B (SsnB) is a potent TLR-4 antagonist, that shows promise as a pharmacological therapy in inflammatory conditions (Yepuri et al., 2019). Despite its potency as a TLR-4 antagonist, SsnB’s flat aromatic structure and poor aqueous solubility leads to significantly reduced drug bioavailability (Liang et al., 2015). One study shows the absolute bioavailability of SsnB is only approximately 7% (Zou et al., 2015), thus restricting its therapeutic applications. The current study uses a nano-formulation of SsnB (nSsnB) formulated with a linear PEGylated dendritic telodendrimer PEG^5k^CA_4_Rf_4_ to increase SsnB solubility and bioavailability. Our results provide evidence that nSsnB is less toxic and can ameliorate inflammation in LPS-treated RAW264.7 cells. Prophylactic intratracheal (IT) nSsnB also attenuates systemic inflammation and lung injury in ALI mouse model induced by the IT LPS administration. Our results also provide evidence that nSsnB downregulates inflammation and improves lung injury by inhibiting pulmonary MyD88/NF-κB activation.

## 2 Materials and methods

### 2.1 The development of SsnB nano-formulation

The telodendrimer PEG^5k^CA_4_Rf_4_ was used to encapsulate SsnB. For nomenclature, PEG^5k^ represents a polyethylene glycol with a molecular weight of 5 kDa and CA_4_Rf_4_ indicates that four cholic acids and riboflavin molecules were conjugated on α-amino and ε-amino groups respectively on the periphery of dendritic polylysine. PEG^5k^CA_4_Rf_4_ was prepared following our previous procedure via peptide chemistry (Guo et al., 2017; Shi et al., 2015). SsnB was encapsulated within a telodendrimer (TD) nanocarrier, PEG^5k^CA_4_Rf_4_, using a thin-film hydration method as previously described (Guo et al., 2017; Shi et al., 2015). Briefly, SsnB solution in DMSO was added into TD solution in CHCl_3_/MeOH (10:1 v/v) mixture at a TD/SsnB weight ratio of 10:1 (w/w). The solvents were evaporated on a Rota evaporator, resulting in a thin, homogeneous film of SsnB-TD mixture coated on the flask wall. The film was further dried under high vacuum for 1 h. Subsequently, the film was hydrated in PBS. The particle size distributions of the drug-loaded nanoparticles (NPs) were determined using dynamic light scattering (DLS) Zetasizer (Malvern).

### 2.2 Cell culture and effect of SsnB on cell viability and inflammation in RAW264.7 cells

The RAW264.7 macrophage cell line was obtained from American Type Culture Collection (Rockville, MA). Cells were maintained in Dulbecco’s Modified Eagle’s Medium (Gibco, Gaithersburg, MD) and supplemented with 10% fetal bovine serum (Atlanta Biologicals, Flowery Branch, GA), 1 mM sodium pyruvate (Gibco) and 100 U/mL penicillin G and 100 μg/mL streptomycin at 37°C in a humidified incubator with 5% CO_2_ (Meng et al., 2024; Guo et al., 2024). The growth medium was refreshed every other day. A phase contrast microscope was used to monitor the RAW264.7 cells daily.

For cell viability study, Raw264.7 cells were seeded in a 96-well plate with the cell density of 4 × 10^3^ cells per well. After an overnight incubation, the cells were treated with different concentrations of free SsnB or nSsnB, and blank TD. After 72 h incubation, Cell titer 96 aqueous cell proliferation reagent, which is composed of 3-(4,5-dimethylthiazol-2-yl)-5-(3-carboxymethoxyphenyl)-2-(4-sulfophenyl)-2H tetrazolium (MTS, Cat. #: G3582, Promega) and an electron-coupling reagent phenazine methosulphate (PMS), was added to each well according to the manufacturer’s instructions and further incubated for 1–4 h at 37°C. The cell viability was determined by measuring the absorbance at 490 nm using a microplate reader (BioTek Synergy H1). Untreated cells served as a control. Results were shown as the average cell viability of triplicate wells via an equation: Cell viability % = [(OD_treat_-OD_blank_)/(OD_control_-OD_blank_) × 100%].

For examining the effect of nSsnB on inflammation, RAW264.7 6-well plate with the cell density of 3 × 10^5^ cells per well. After an overnight incubation, the cells were treated with LPS (1 μg/mL) ± nSsnB (32 μg/mL) for 24 h. The culture medium and cells were collected for detection of inflammatory cytokines and protein analysis, respectively.

### 2.3 Hemolytic assays

Fresh blood was obtained from a healthy human volunteer and mixed with a PBS solution containing 20 mM EDTA. Red blood cells (RBCs) were then isolated by centrifugation at 1,000 rpm for 10 min. Subsequently, the RBCs were washed three times with PBS and resuspended in PBS (Guo et al., 2024; Guo et al., 2020; Wang et al., 2019). Free SsnB or nSsnB were added to 200 µL of RBC solutions at concentrations of 10, 100, and 200 μg/mL, followed by gentle mixing and incubation at 37°C for 30 min, 4 h, and overnight, respectively. Afterward, the samples were centrifuged at 1,000 rpm for 5 min, and the hemoglobin in the supernatant was measured using a NanoDrop spectrophotometer at 540 nm. PBS and Triton X-100 (2%) were included as negative and positive controls, respectively, by incubating them with RBCs. Hemolytic toxicity was determined using the following equation: Hemolysis% = [(OD_sample_ - OD_PBS_)/(OD_triton_ - OD_PBS_)] × 100%.

### 2.4 Animals and lung injury model

Male and female C57BL/6 and HIV-LTR/Luciferase (HLL) mice, aged 8 weeks, were purchased from Jackson Laboratories (Bar Harbor, ME). The mice were housed in a controlled environment with a temperature of 22°C and a photoperiod of 12 h light and 12 h dark, with ad libitum access to food and water. All animal procedures were approved by the Institutional Animal Care and Use Committee of SUNY Upstate Medical University (IACUC #344 and #437) and conducted in compliance with the National Institutes of Health and ARRIVE guidelines for the use of laboratory animals.

### 2.5 LPS-induced lung injury, nSsnB treatment and tissue harvest

All mice received non-invasive tracheal instillation using an aerosolizer with nSsnB (0.625 mg/kg) or vehicle 2 h prior to inducing lung injury with LPS (2.5 mg/kg) or saline, as previously described (Ortiz-Munoz and Looney, 2015; Meng et al., 2022). Briefly, mice in the septic and control groups were anesthetized via intraperitoneal injection of a combination of ketamine (80 mg/kg) and xylazine (8 mg/kg). After induction of anesthesia, mice were positioned in the supine position on an intubating platform, with their incisors suspended and a fiber-optic illuminator positioned over the trachea. Tongue retraction and upward-leftward positioning were performed using forceps to visualize the larynx, aided by hands-free binocular magnifiers. The MicroSprayer Aerosolizer (Cat. #: YAN30012, Shanghai Yuyan Instruments Co., Ltd.) was then inserted into the tracheal lumen, and LPS, nSsnB, and saline solution (not exceeding a volume of 70 µL per mouse) were instilled. Mice were maintained in the same position on the intubating platform for at least 30 s before being placed prone on a heating pad for recovery. All surviving mice were euthanized under anesthesia at 24 h post-LPS administration, and blood (in EDTA), lung tissue (fixed with 10% formalin for histology and frozen for protein analysis), and bronchoalveolar lavage fluid (BALF) were collected. The IT administration of LPS (2.5 mg/kg) in this study did not result in mortality among any of the experimental groups.

### 2.6 Cytological and protein analysis in BALF

BALF was collected from the mouse lung and lavaged with three aliquots of 0.5 mL sterile saline, followed by centrifugation at 250 × g for 10 min. The resulting pellet was resuspended in 1 mL of sterile saline. A 100 µL portion of the cell suspension was then centrifuged using a cytospin centrifuge (Hettich ROTOFIX 32A) at 1,000 rpm for 3 min to adhere the cells to a slide (Meng et al., 2022). The slide was air-dried and stained with Hema-3 (Cat. #: 23-122929, 23-122937, 23-122952, Fisher Scientific, Kalamazoo, MI) for subsequent analysis. Neutrophils and macrophages were quantified in 20 high-power fields (HPF) by blinded reviewers utilizing a Nikon Eclipse TE2000-U research microscope (Nikon, Melville, NY). Protein concentration in BALF was measured using the BCA micro assay kit from Thermo Scientific (Cat. #: 23235) according to the provided guidelines.

### 2.7 Western blot and ELISA

Lung tissue was homogenized in RIPA buffer, and the extracted protein was utilized for Western blot analysis (Meng et al., 2023). A total of 20 µg of protein was separated by SDS-PAGE gel electrophoresis, followed by transfer to PVDF membranes (Cat. #: IPVH00010, Millipore Co., Ltd. United States). The membranes were blocked with 5% non-fat milk (Cat. #: 1706404XTU, Bio-Rad Laboratories) in Tris-buffered saline plus 0.5% Tween-20 (TBS-T) from Fisher Scientific (Cat. #: BP337-500) for 1 h at room temperature, and then incubated overnight at 4°C with primary antibodies purchased from Santa Cruz Biotechnology, including MyD88 (Cat. #: sc-74532, 1:200 dilution) and phosphorylated-NF-κB (p65) (Cat. #: sc-136548, 1:200 dilution). Subsequently, the membranes were probed with a secondary antibody conjugated to horseradish peroxidase (HRP) obtained from Santa Cruz Biotechnology (Cat. #: 516102, 1:1,000 dilution) for 1 h at room temperature. Antibody-antigen complexes were visualized using enhanced chemiluminescence (ECL, Cat. #: 34580, Thermo Scientific) according to the manufacturer’s instructions. Quantitative analysis of the immunoreactive bands was performed using densitometry with ImageJ software, and the relative density of the bands was normalized to the density of the corresponding β-actin bands.

Blood samples, BALF and lung tissue were collected to measure levels of TNF-α (Cat. #: 50-112-8800, Invitrogen) and IL-6 (Cat. #: 50-112-8863, Invitrogen) by ELISA. Serum from blood and BALF were used directly for ELISA following appropriate dilution. Lung tissue required homogenization in protein lysis buffer (RIPA) to extract the supernatant, followed by protein concentration measurement using a BCA assay. The protein solution then was diluted to a final concentration of 1 mg/mL before ELISA analysis. All cytokines were quantified using commercial ELISA kits in accordance with the manufacturer’s instructions.

### 2.8 Histopathological evaluation of lung injury

The lungs were fixed for histological examination by instilling 0.5 mL of 10% neutral formalin through the trachea. Following fixation, the lungs were embedded in paraffin, and 5 μm sections were prepared for staining with Hematoxylin and Eosin (H&E). Histopathological analysis was conducted in a blinded manner by two independent pathologists. Assessment of acute lung injury was performed using a scoring system ranging from 0 to 2, as described in a previous study (Matute-Bello et al., 2001; Meng et al., 2022). This scoring system evaluated parameters such as neutrophil presence in the alveolar and interstitial spaces, hyaline membrane formation, proteinaceous debris in airspaces, and septal thickening. A lung injury score was calculated by summing the scores of each parameter, which were then normalized to the number of fields evaluated. Three representative fields per slide were assessed in 20 high-power fields (HPF) by blinded reviewers using light microscopy at ×400 magnification.

### 2.9 *In vivo* measurement of luciferase gene expression by bioluminescence imaging with HLL mice

HIV-LTR/Luciferase (HLL) mice are genetically modified mice that are used to study the regulation of gene expression IVIS, particularly in response to NF-κB-mediated signaling, as luciferase activity can serve as a reporter for the activation of this pathway (Blackwell et al., 2000). All bioluminescent data were collected and analyzed using *in vivo* Imaging Systems (IVIS) at Upstate medical university core research facilities.

### 2.10 Statistical analysis

The data were expressed as either mean ± SEM or median ± interquartile range following tests for normal distribution using SPSS 22 (SPSS, Chicago, United States). The sample size for each experimental group (n = 3–7) is indicated in the figure legend. Group differences were assessed using either one-way analysis of variance (one-way ANOVA) with Bonferroni’s multiple comparisons test (for normally distributed data) or Kruskal-Wallis test with Dunn’s multiple comparison test (for non-normally distributed data). Statistical significance was considered at P < 0.05. All data were obtained from three or more independent experiments.

## 3 Results

### 3.1 *In vitro* characterization of SsnB nanoformulation

**FIGURE 1:**
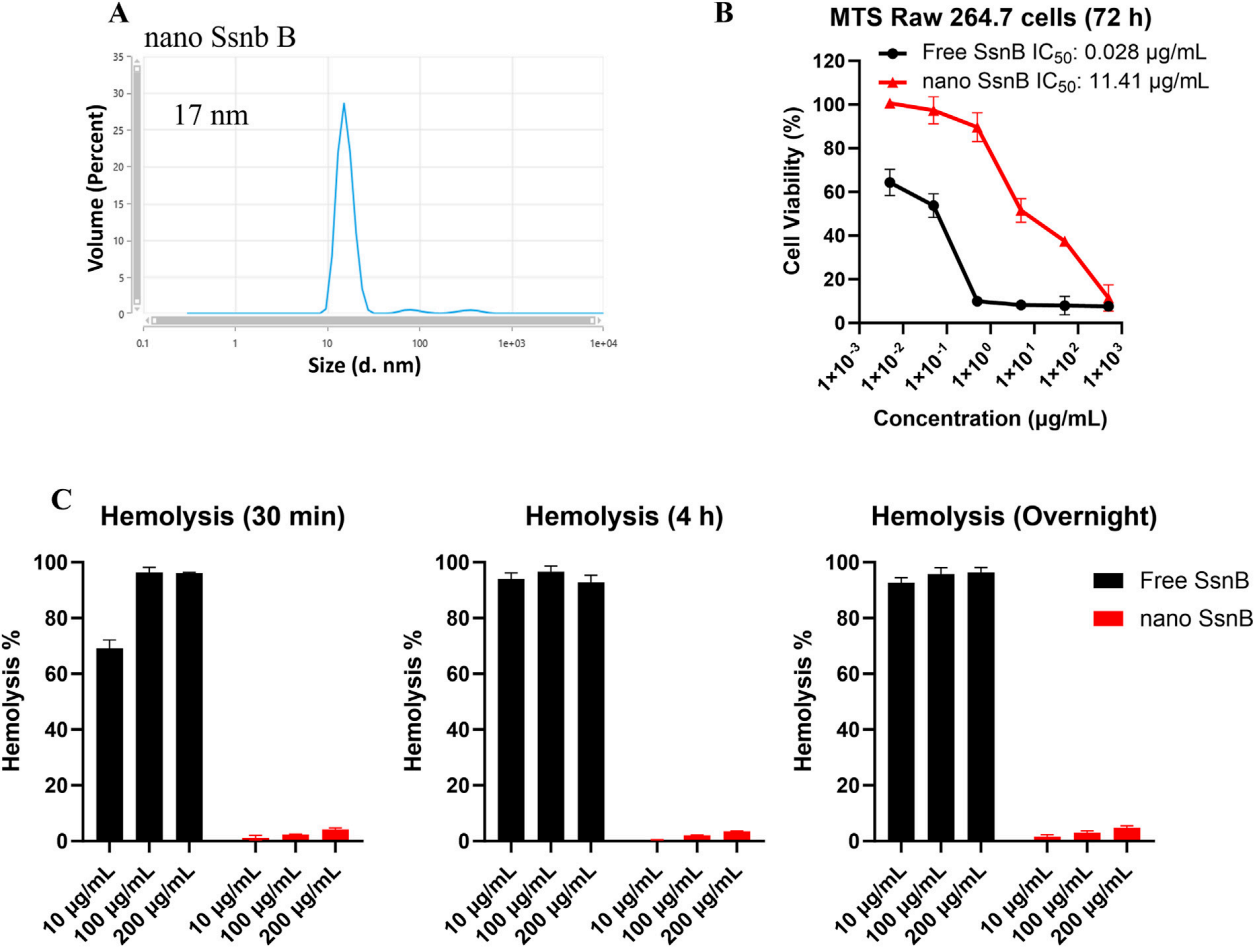
Characterization of nano SsnB (nSsnB) and analysis of cell viability and hemolytic toxicity. **(A)** Particle size of nano SsnB obtained by DLS. **(B)** Cell viability of RAW 264.7 cells treated with free SsnB and nSsnB for 72 h. **(C)** Hemolytic toxicity of free SsnB and nSsnB incubated with red blood cells at 37°C for 0.5 h, 4 h and overnight.

**FIGURE 2:**
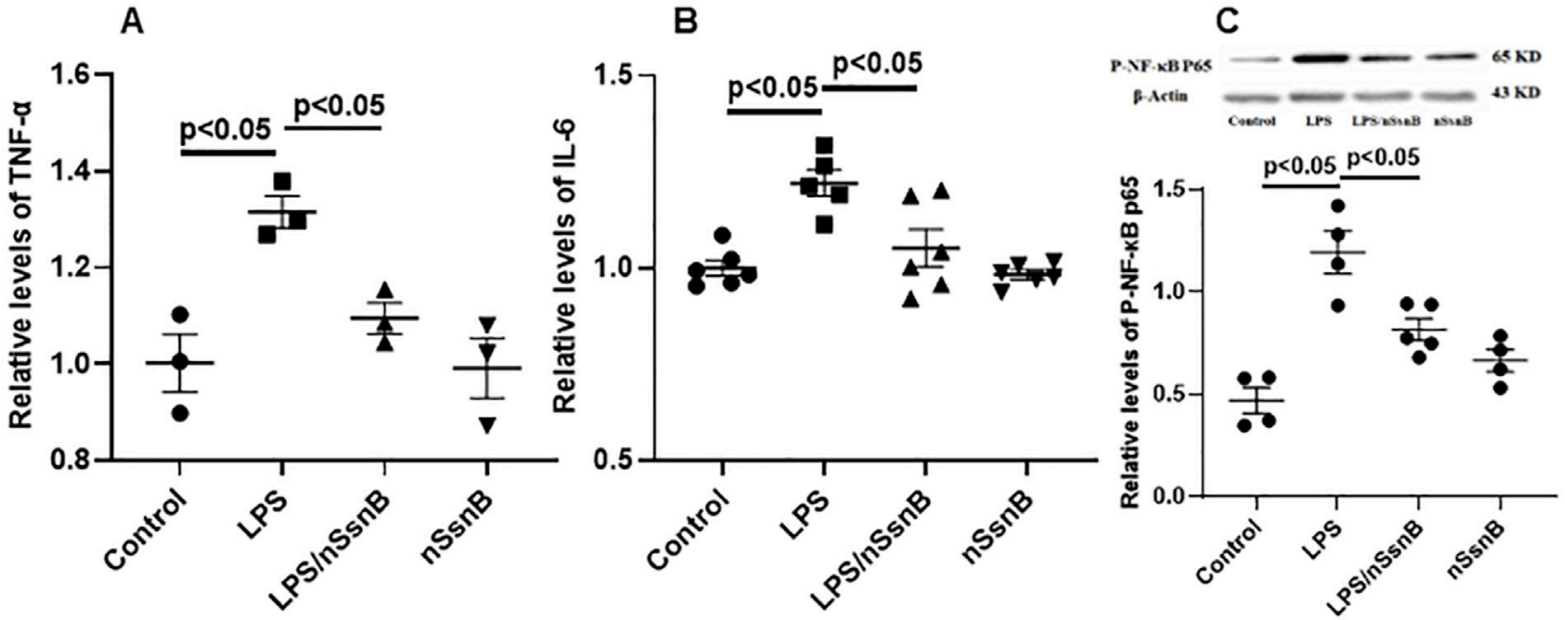
The effect of nano-formulation of SsnB (nSsnB) on inflammation and NF-κB signaling in LPS-treated Raw264.7 cells. Raw264.7 cells were treated with LPS (1 μg/mL) and nSsnB (32 μg/mL) for 24 h. The media were collected for the measurements of inflammatory mediators by ELISA. TNF-α **(A)** and IL-6 **(B)** were assayed to assess the severity of inflammation. Cell lysate was isolated for protein and Western blot was performed and normalized to β-actin to examine the levels for protein expression. The representative images of phosphorylated NF-κB P65 (p-NF-κB P65) and the corresponding quantitative results were shown in **(C)**. Representative Western blot images are presented in Figure 2C, with additional images available in the Supplementary File 1. Scatter dot plot represents mean values and standard error of mean (SE) (n = 3–6/group).

Given the ultra flat aromatic structure of SsnB, we have screened our telodendrimers with aromatic building blocks as reported in our previous study (Shi et al., 2015) for SsnB encapsulation. Majority of TD nanocarriers fail to encapsulate SsnB, either for poor loading capacity or stability. As a result, PEG^5k^CA_4_Rf_4_ showed a stable SsnB loading, owing to the aromatic riboflavin building block. In addition, both cholic acid (CA) and riboflavin are amphiphilic, which dramatically stabilize drug loading and nanoparticle stability. As shown in Figure 1A, nSsnB has a uniform and small particle size around 17 nm, which is stable upon storage for weeks. Although SsnB was reported to be a potent TLR4/MD2 inhibitor, it was also shown to have anticancer (Liu et al., 2021) and antiangiogenic (Bateman et al., 2013) activity. Indeed, free SsnB showed significant cytotoxicity upon murine macrophage cell line Raw 264.7 in cell culture with an IC50 of 28 ng/mL (Figure 2B), which is significantly reduced by nSsnB for about 500 fold to 11.4 ug/mL. At the same time, free SsnB has very high hemolytic properties in the *in vitro* whole blood incubation with almost instant hemolysis after 30 min incubation at 10 ug/mL. While nSsnB is safe without significant hemolysis even overnight incubation at 200 ug/mL (Figure 1C).

We further examined the anti-inflammatory effect of nSsnB in immune cell culture (RAW264.7) in the presence of LPS co-incubation. LPS significantly induced cytokine productions in the culture medium as detected by ELISA, e.g., TNF-α (Figure 2A) and IL-6 (Figure 2B), in comparison to the control. The nSsnB significantly inhibited cytokine productions induced by LPS. And it was shown that nSsnB does not have an immunostimulatory effect without cytokine induction. We further examined the activation of NF-κB in immune cells with Western blot assay. As shown in Figure 2C, the phosphorylated NF-κB P65 (p-NF-κB P65) (P < 0.05 vs. Control) induced by LPS can be effectively inhibited by nSsnB. While nSsnB had no effects on this inflammatory signaling in the comparison to the control group. These results clearly demonstrated that nSsnB possesses an anti-inflammatory effect by inhibiting the LPS-TLR-4/NF-κB pathway.

### 3.2 Effects of nSsnB on LPS-induced systemic inflammation and ALI

**FIGURE 3:**
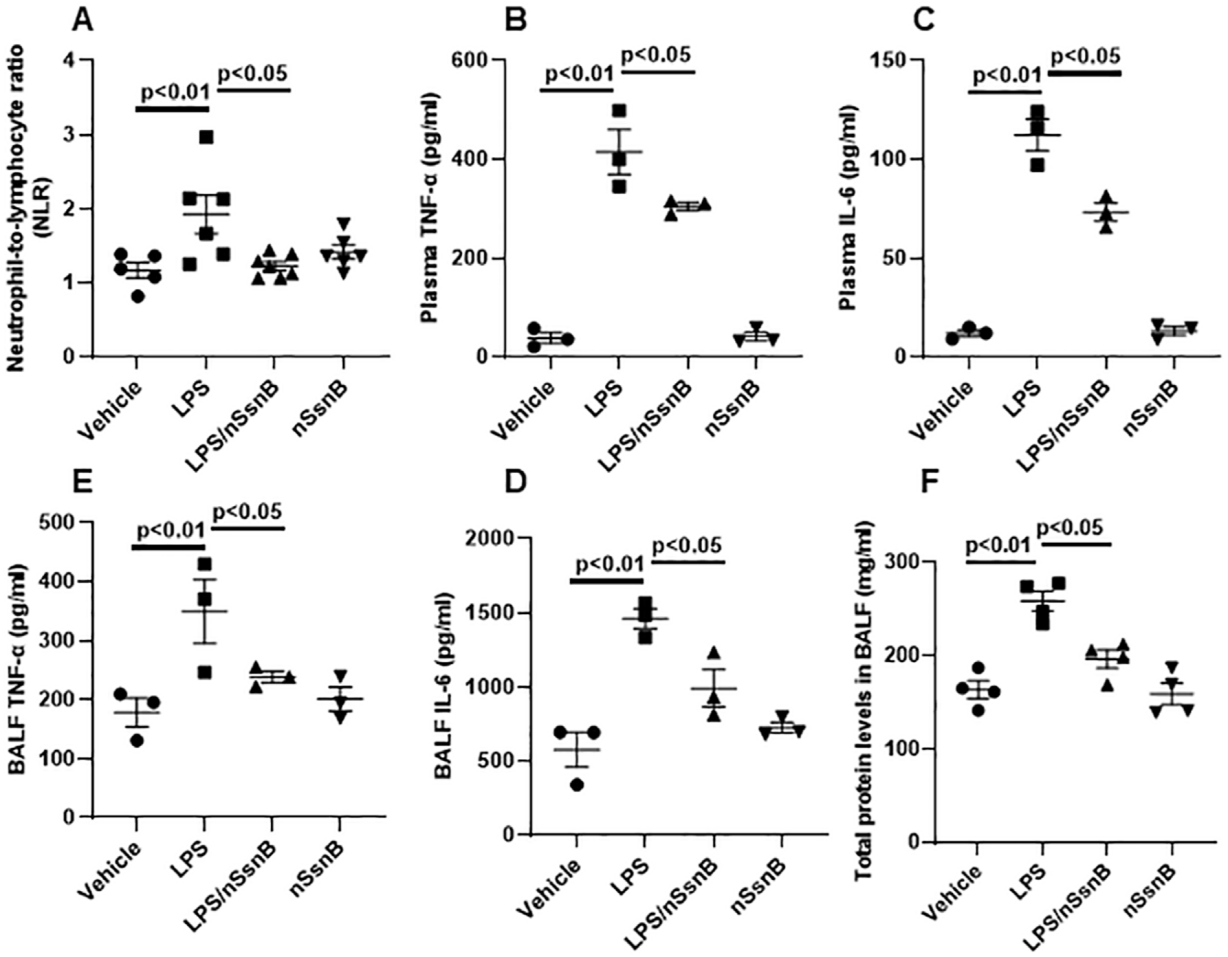
Hematological analysis in blood, and cytokine analysis in plasma and bronchoalveolar lavage fluid (BALF) as wells as protein analysis in BALF from C57BL/6J mice: Mice were treated with nSsnB (0.625 mg/kg) or vehicle 2 h after induction of lung injury by LPS (2.5 mg/kg) or sham lung injury (by saline) using noninvasive tracheal installation by aerosolizer. Mice were sacrificed 24 h after nSsnB or saline, then blood/plasma and BALF were collected for blood cell counting and cytokines by ELISA. Protein was assayed using BCA. Neutrophil-to-lymphocyte ratio (NLR%) **(A)** were calculated. TNF-a **(B, D)** and IL-6 **(C, E)** in plasma and BALF were assayed. Total protein in BALF was shown in **(F)**. Box-and-Whisker Plot represents median and interquartile interval (n = 4–6/group).

Assessment of the neutrophil-to-lymphocyte ratio (NLR) in blood during LPS-induced lung injury permits investigation of the inflammatory response and immune cell dynamics. By measuring changes in NLR, we can gain insights into the balance between pro-inflammatory (neutrophils) and anti-inflammatory (lymphocytes) responses in the blood circulation following exposure to LPS. The LPS-induced increase in NLR was attenuated by nSsnB (Figure 3A). Consistent with these findings, the LPS-induced increases in plasma TNF-α (Figure 3B) and IL-6 (Figure 3C) were both ameliorated by prophylactic nSsnB administration. Collectively these findings suggest that prophylactic nSsnB ameliorates LPS-induced systemic inflammation.

We measured inflammatory cytokine levels in BALF as an indicator if lung inflammation and total BALF protein as an indicator of increased vascular permeability in ALI. The LPS-induced increase in BALF TNF-α (Figure 3D) and IL-6 (Figure 3E) were significantly reduced by nSsnB (P < 0.05, LPS vs. LPS/nSsnB). Likewise, the increased BALF protein levels observed in LPS-treated mice were significantly reduced by prophylactic nSsnB (Figure 3F). Collectively these finding suggests nSsnB attenuates both systemic and pulmonary inflammation in this model.

### 3.3 BALF cytology

**FIGURE 4:**
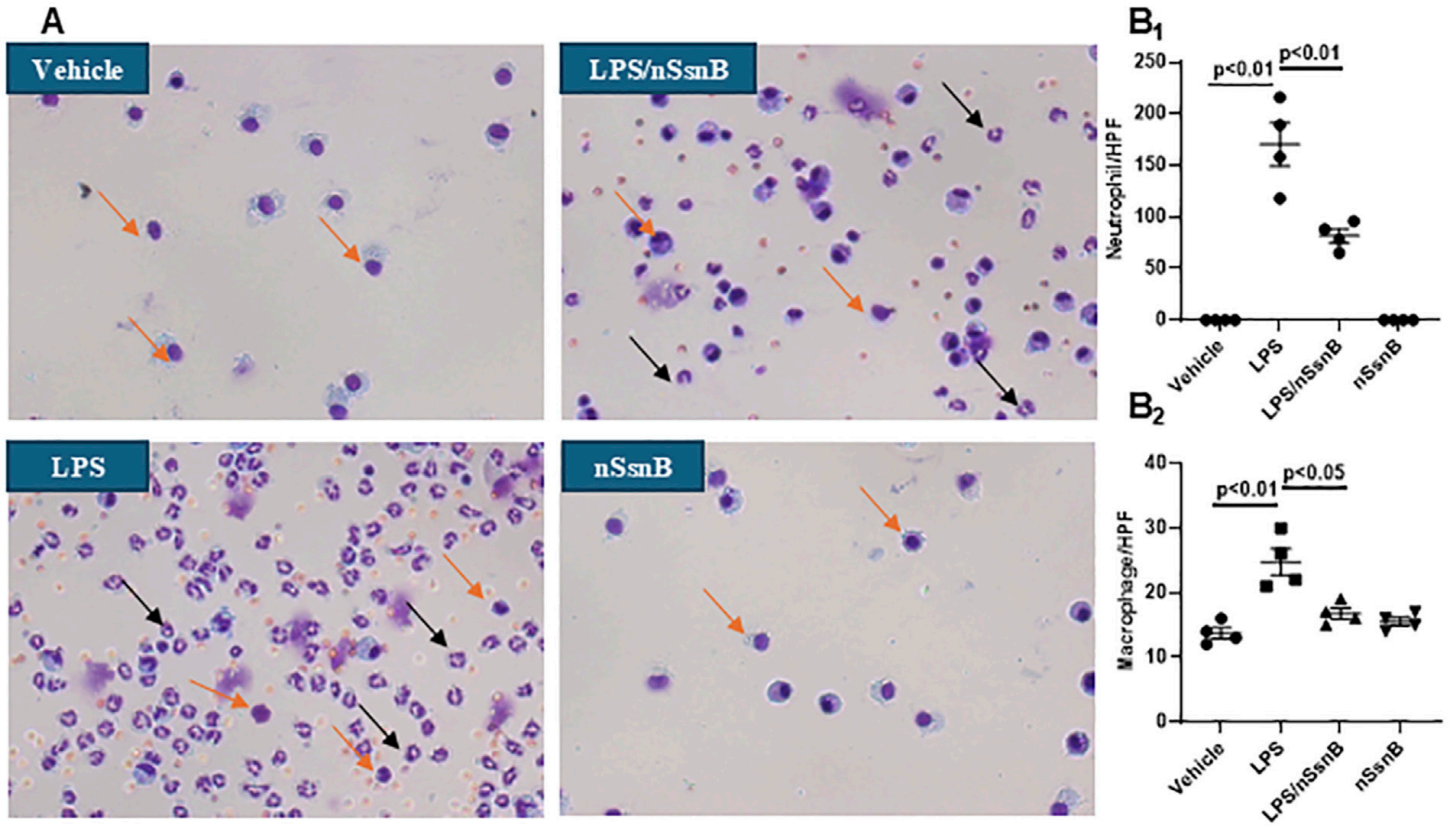
Cytological analysis in BALF: C57BL/6J mice were treated with nSsnB (0.625 mg/kg) or vehicle 2 h after induction of lung injury by LPS (2.5 mg/kg) or sham lung injury (by saline) using noninvasive tracheal installation by aerosolizer. Mice were sacrificed 24 h after nSsnB or saline, then BALF was collected for examining neutrophils (black arrows) and macrophages (yellow arrows) by Hema three staining **(A)**. Quantification of neutrophils **(B**_**1**_ **)** and macrophages **(B**_**2**_ **)** per slide were counted at 400 magnifications under light microscopy. Box-and-Whisker Plot represents median and interquartile interval (n = 3–7/group).

Lung injury and inflammation are characterized by increased leukocytes in the BALF. Hema-3 staining was used for cytological analysis of BALF (Figure 4A). Neutrophils (Figure 4B_1_) and macrophages (Figure 4B_2_) were both increased in BALF from LPS-treated mice. In contrast, pretreatment with nSsnB significantly reduced both Neutrophils and macrophages in the LPS/nSsnB group (P < 0.05, LPS vs. LPS/nSsnB). The reductions in BALF leukocytes observed in this experiment support the anti-inflammatory effects of nSsnB on LPS-induced lung inflammation and injury.

### 3.4 Analysis of lung histology in LPS-induced ALI in C57BL/6 mice

**FIGURE 5:**
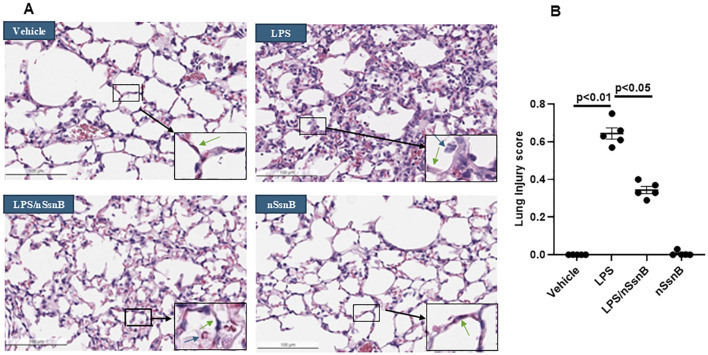
Histological assessment of lung injury: C57BL/6J mice were treated with nSsnB (0.625 mg/kg) or vehicle 2 h after induction of lung injury by LPS (2.5 mg/kg) or sham lung injury (by saline) using noninvasive tracheal installation by aerosolizer. Mice were sacrificed 24 h after nSsnB or saline, then lung tissue was collected for H&E staining to evaluate lung injury from each group **(A)**. Lung injury was characterized by neutrophil infiltration (blue arrows), hyaline membranes, proteinaceous debris filling the airspaces and alveolar septal thickening (green arrow). Semi-quantitative histological lung injury score was assessed **(B)**. Box-and-Whisker P.lot represents median and interquartile interval (n = 5/group).

Analysis of lung histology is critical to assessing the severity of lung injury, validating animal models, and evaluating potential therapies. LPS-induced lung injury is characterized by polymorphonuclear neutrophils (PMNs) infiltration, alveolar hemorrhage, edema, and the formation of hyaline membranes. Pre-treatment with nSsnB mitigated histological lung injury (Figure 5A) and reduced the lung injury score (Figure 5B) in LPS-induced lung injury (P < 0.05, LPS vs. LPS/nSsnB). The histological findings support the therapeutic efficacy of prophylactic nSsnB in attenuating LPS-induced lung injury.

### 3.5 Pulmonary activation of MyD88 and NF-κB

**FIGURE 6:**
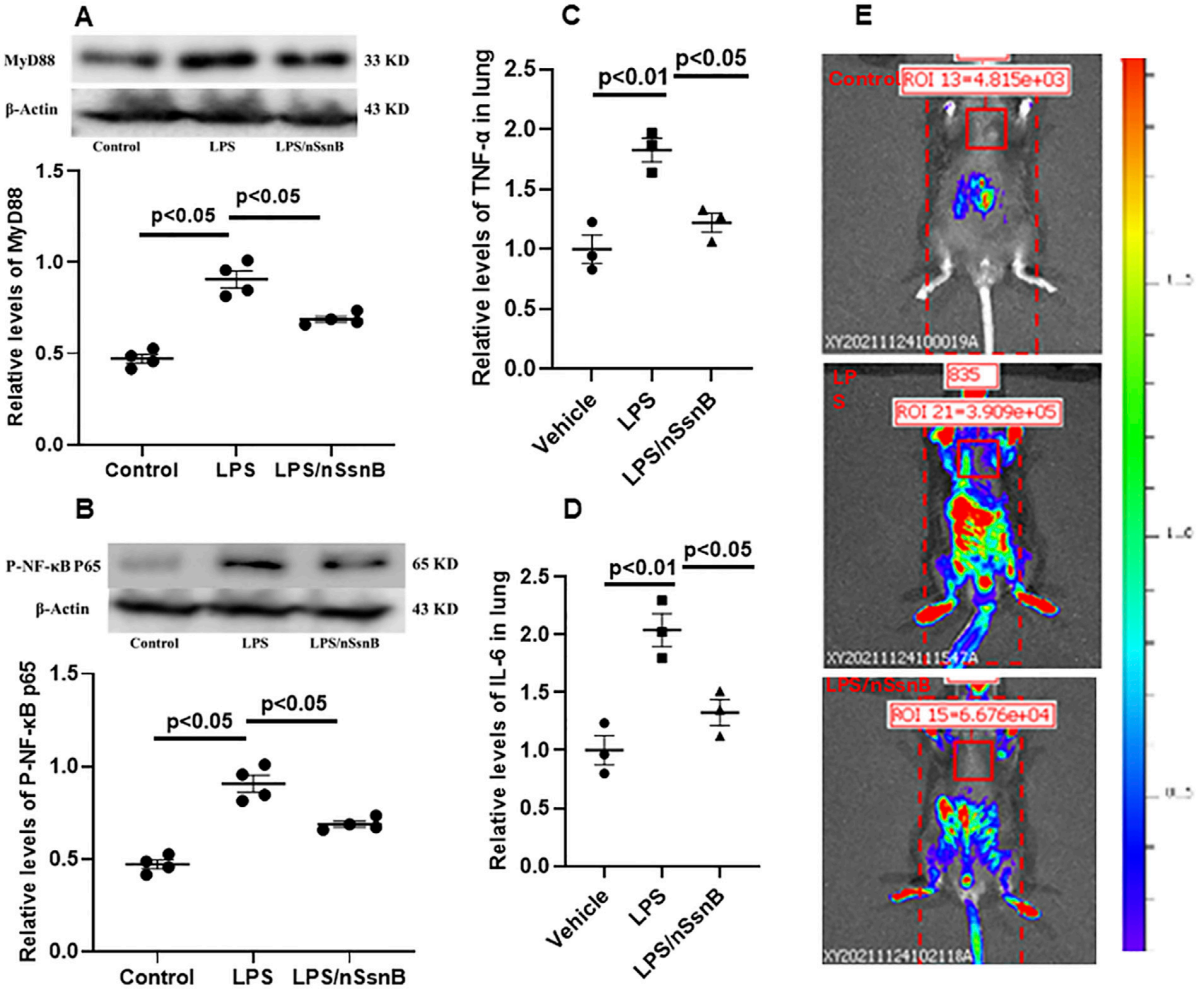
The measurement of MYD88/NF-κB activation in C57BL/6J and HIV-LTR/Luciferase (HLL) mice. Mice were treated with nSsnB (0.625 mg/kg) or vehicle 2 h after induction of lung injury by LPS (2.5 mg/kg) or sham lung injury (by saline) using noninvasive tracheal installation by aerosolizer. C57BL/6J mice were sacrificed 24 h after nSsnB or saline, then lung tissue was collected for protein analysis. Western blot was performed and normalized to β-actin to examine the levels for protein expression. The representative images of MYD88 and phosphorylated NF-κB P65 (p-NF-κB P65) and the corresponding quantitative results were shown in **(A, B)**. Cytokine levels of TNF-α and IL-6 in lung tissues were presented in **(C, D)**. The HLL mice act as a luciferase reporter for NF-κB activation and inflammatory responses. Bioluminescence level was acquired by *In Vivo* Imaging Systems (IVIS) 24 h after nSsnB or saline. Representative images representing activation of NF-κB were shown in 6E.Representative Western blot and *In Vivo* images are presented in Figure 6, with additional images available in the Supplementary File 1. Scatter dot plot represents mean values and standard error of mean (SE) (n = 4/group).

Myeloid Differentiation Primary Response 88 (MyD88) and Nuclear Factor-kappa B (NF-kB) are crucial components of the Toll-like receptor (TLR) signaling pathway, especially in the context of TLR-4 activation. LPS significantly increased the pulmonary levels MyD88 (Figure 6A) and p-NF-κB P65 (Figure 6B) in C57BL/6 mice (P < 0.05 vs. Control). However, pretreatment with nSsnB significantly reduced the LPS-induced increases in both MyD88 and p-NF-κB P65 (P < 0.05 vs. LPS). LPS-induced activation of NF-κB p65 drives the transcription of pro-inflammatory cytokines, including TNF-α (Figure 6C) and IL-6 (Figure 6D). Our results demonstrate that the elevated levels of TNF-α and IL-6 in lung tissue were significantly attenuated by nSsnB treatment. We also examined pulmonary NF-κB activation using HLL mice to investigate the effects of LPS/nSsnB on NF-κB signaling *in vivo* (Figure 6E). The signal intensity was significantly elevated in the HLL LPS-treated mice compared to controls. As expected, pretreatment with nSsnB markedly reduced the LPS-induced signal enhancement. Overall, our *in vivo* and *in vitro* data validate SsnB as an inhibitor of TLR-2/4 signaling by blocking MyD88 and NF-κB, leading to reductions in inflammation and lung injury.

## 4 Discussion

ARDS is a potentially life-threatening complication of sepsis, severe trauma (indirect), pneumonia, aspiration of gastric contents, or inhalation injuries (direct). Rapidly progressive respiratory failure due to ARDS accounts for 10% of ICU admissions and has a mortality rate of 40% in severe cases (Ramji et al., 2023). Although supportive care including antibiotics, vasopressors, mechanical ventilation and renal replacement therapy improves outcomes, many patients develop multiple organ failure despite these aggressive treatments. Although inflammation is part of the normal host response to sepsis, severe systemic inflammation or an exaggerated inflammatory response are felt to be important in the pathogenesis of ARDS and multiple organ failure. The current study examines the anti-inflammatory effects of prophylactic nSsnB on inflammation in cultured macrophages and LPS-induced lung injury in mice. Recent studies suggested nanomedicines have been applied in respiratory diseases treatment, like chronic obstructive pulmonary disease and asthma (Afzal et al., 2022; Prakash, 2023; Pandey et al., 2022; Sadikot et al., 2017; Prasanna et al., 2021). SsnB is a flat aromatic molecule with poor solubility in water as well as many organic solvents. DMSO is needed to dissolve it for loading and bioactivity studies. We purposely introduce flat aromatic riboflavin into telodendrimer PEG^5k^CA_4_Rf_4_ to increase molecular interaction with SsnB for efficient encapsulation. In addition, the facial amphiphilic cholic acid (CA) was introduced in TD to further stabilize SsnB encapsulation by reducing the surface energy between aqueous shell and hydrophobic core of nanoparticle. As a result, the nano-formulation of SsnB used in our study (nSsnB) enhances its water solubility for feasible *in vivo* application.

In previous studies, SsnB was solubilized in dimethyl sulfoxide (DMSO) or polyethylene glycol, with concentrations significantly higher than those used in our study (0.625 mg/kg for mouse). Administration routes were predominantly oral (300 mg/kg for animal study) (Yuan et al., 2018) or via intraperitoneal injection (3–9 mg/kg for animal study) (Dattaroy et al., 2016; Dattaroy et al., 2018; Liang et al., 2015; Ma et al., 2022; Wang et al., 2018). Notably, pretreatment was conducted over a treatment period ranging from 2 to 28 days. To facilitate comparison with prior research, our current study maintained the practice of administering the nSsnB before lung injury but differed by using a single dose via intratracheal delivery. Despite lower doses and intratracheal delivery, our data provide evidence that nanoformulated SsnB has significant anti-inflammatory effects and attenuates LPS-induced lung injury.

The toxicity and safety profile of SsnB have been documented in prior investigations (Liang et al., 2015). Following intraperitoneal injection of different doses of SsnB (dissolved in DMSO), mice were monitored for 6 days. None of the mice, even those administered the highest dose of SsnB (500 mg/kg), exhibited any evident abnormalities or fatalities. Additionally, histological examination did not reveal any discernible alterations in organs and tissues, including the lung, liver, spleen, and intestine (Liang et al., 2015). Our results showed that both the nanoformulation of SsnB and polymer alone demonstrated no impact on cell viability in RAW264.7 cells. These data suggest our nSsnB preparation is safe for use within the dose range used in our experiments.

Our results provide evidence that nSsnB mitigates inflammation in cultured macrophages by attenuating NF-κB activation and cytokine production. nSsnB also attenuates LPS-induced lung injury by attenuating activation of the TLR-4/MyD88/NF-κB signaling pathway. When TLR-4 is activated, MyD88 (an adaptor protein) is recruited to the TLR cytoplasmic domain, forming a signaling complex that activates downstream signaling cascades. The MyD88-dependent signaling pathway phosphorylates and activates NF-κB, a transcription factor that stimulates production of inflammatory cytokines and chemokines. The ability of nSsnB to attenuate LPS-induced increases in pulmonary MyD88 levels, phosphorylated NF-κB levels, inflammatory cytokine production and NF-κB activated luminescence in LPS-treated mice is consistent with SsnB’s ability to block TLR mediated inflammation.

These results not only illustrate the therapeutic potential of inhibiting TLR-4/MyD88/NF-κB pathways in treating ALI/ARDS via intratracheal delivery of nano-formulated SsnB, but also affirm the role of SsnB as a TLR-4 inhibitor.

Our results are consistent with others showing SsnB is a selective TLR-4 antagonist and inhibits intracellular events in TLR-4 signaling (Liang et al., 2011). SsnB has also been shown to attenuate TLR-4-mediated NF-κB activation in a dose-dependent manner, inhibit recruitment of MyD88 to TLR-4 and suppresses LPS-induced increases in TNF-α and IL-1β expression (Liang et al., 2015). These studies also showed that pretreatment with SsnB significantly mitigated lung pathology induced by LPS (Liang et al., 2015). Our findings that nSsnB reduces systemic and pulmonary inflammation, capillary leak and histologic lung injury are consistent with those of (Liang et al. (2015). Our findings add to the growing evidence that the interaction between SsnB and TLR-4 has the capacity to modulate inflammation across diverse conditions, highlighting its potential in the development of novel therapeutic approaches (Yepuri et al., 2019). Literature mining and network analysis unveiled that SsnB targets TLR-4 and MyD88-NF-κB-IL-1β/IL-6/TNF-α pathways, which are significant therapeutic targets in inflammatory diseases (Yang et al., 2024). Recent studies suggest TLR-4 is emerging as a therapeutic target for treating respiratory and neurological complications associated with SARS-CoV-2 infection (Kaushik et al., 2021). Furthermore, it has been suggested that SARS-CoV-2 may bind to and activate TLR4, resulting in increased ACE2 expression, thereby facilitating viral entry and contributing to hyperinflammation (Aboudounya and Heads, 2021).

**FIGURE 7:**
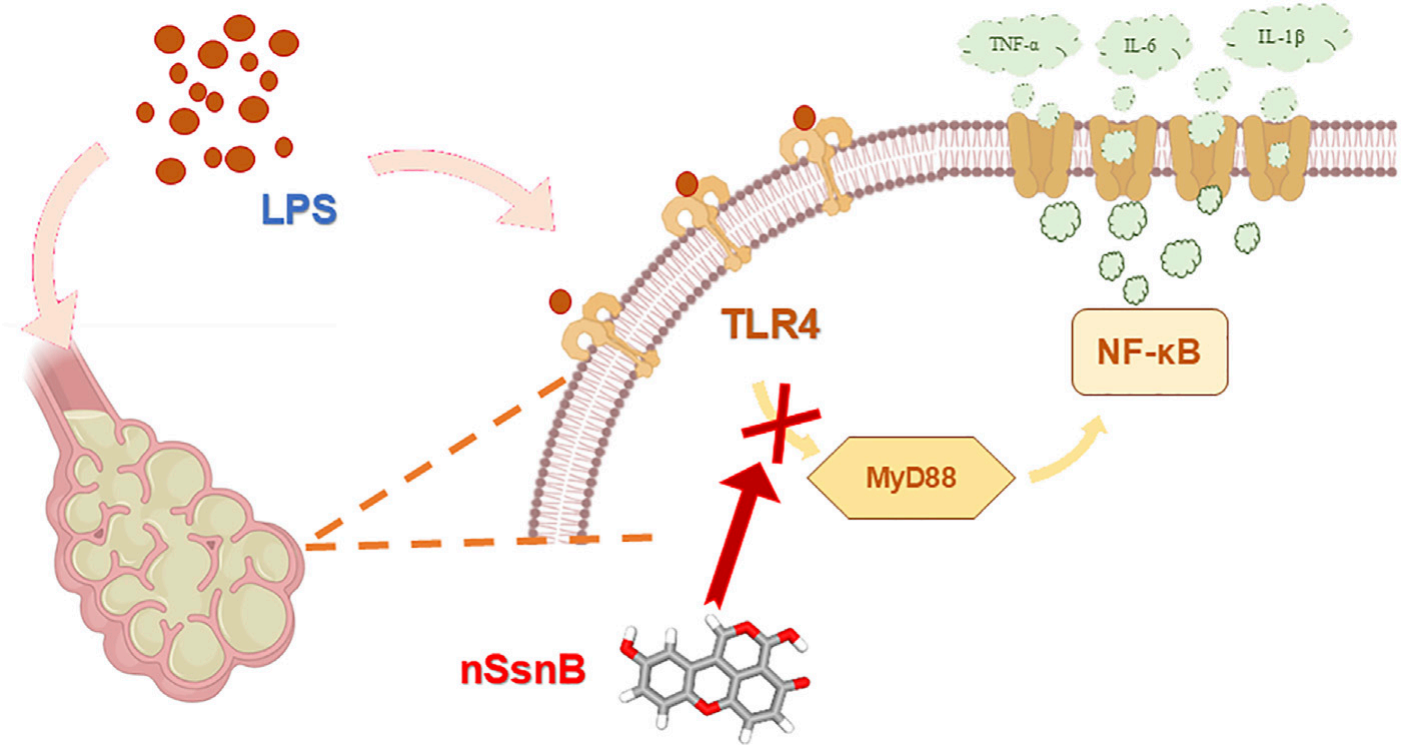
Schematic diagram of the mechanism of action of nSsnB. LPS stimulation activates TLR4, which recruits the adaptor protein MyD88 to initiate downstream signaling. This interaction triggers the activation of NF-κB p65, thereby allowing NF-κB p65 translocation into the nucleus. Nuclear NF-κB promotes the transcription of pro-inflammatory cytokines such as TNF-α and IL-6, amplifying the inflammatory response. Excessive activation of this pathway contributes to lung inflammation and injury. Treatment with nSsnB disrupts TLR4-MyD88 interactions, inhibiting NF-κB activation, reducing cytokine production, and mitigating lung inflammation.

In summary, we report a novel nano-formulation of SsnB which significantly enhances its bioavailability. Prophylactic inhalation of aerosolized nSsnB effectively attenuates LPS-induced inflammation and lung injury by suppressing the MyD88/NF-κB anti-inflammatory pathway. The underlying mechanism and associated signaling pathways are illustrated in detail in Figure 7. While these experimental studies in murine LPS-induced ARDS are promising, it's crucial to acknowledge that findings in animal models may not always directly correlate with human responses. Interspecies variations in lung anatomy, physiology, and immune responses can hinder full reproducibility of results. An additional limitation is that our study omitted measurements of drug pharmacokinetics and tissue biodistribution, although the pharmacokinetics and bioavailability of SsnB have been examined in previous studies involving rats (Zou et al., 2015). Additional studies will be needed to further address these issues.

## Data Availability

The raw data supporting the conclusions of this article will be made available by the authors, without undue reservation.
