# Supplementary material for: Sparstolonin B nano-formulation attenuates LPS-induced lung injury
Source: Front Pharmacol. 2025 Apr 8;16:1532391. doi: 10.3389/fphar.2025.1532391 (PMC12011759; doi:10.3389/fphar.2025.1532391)
Supplement: Supplementary file 1 [file Supplementaryfile1.pptx]

## Slide 1
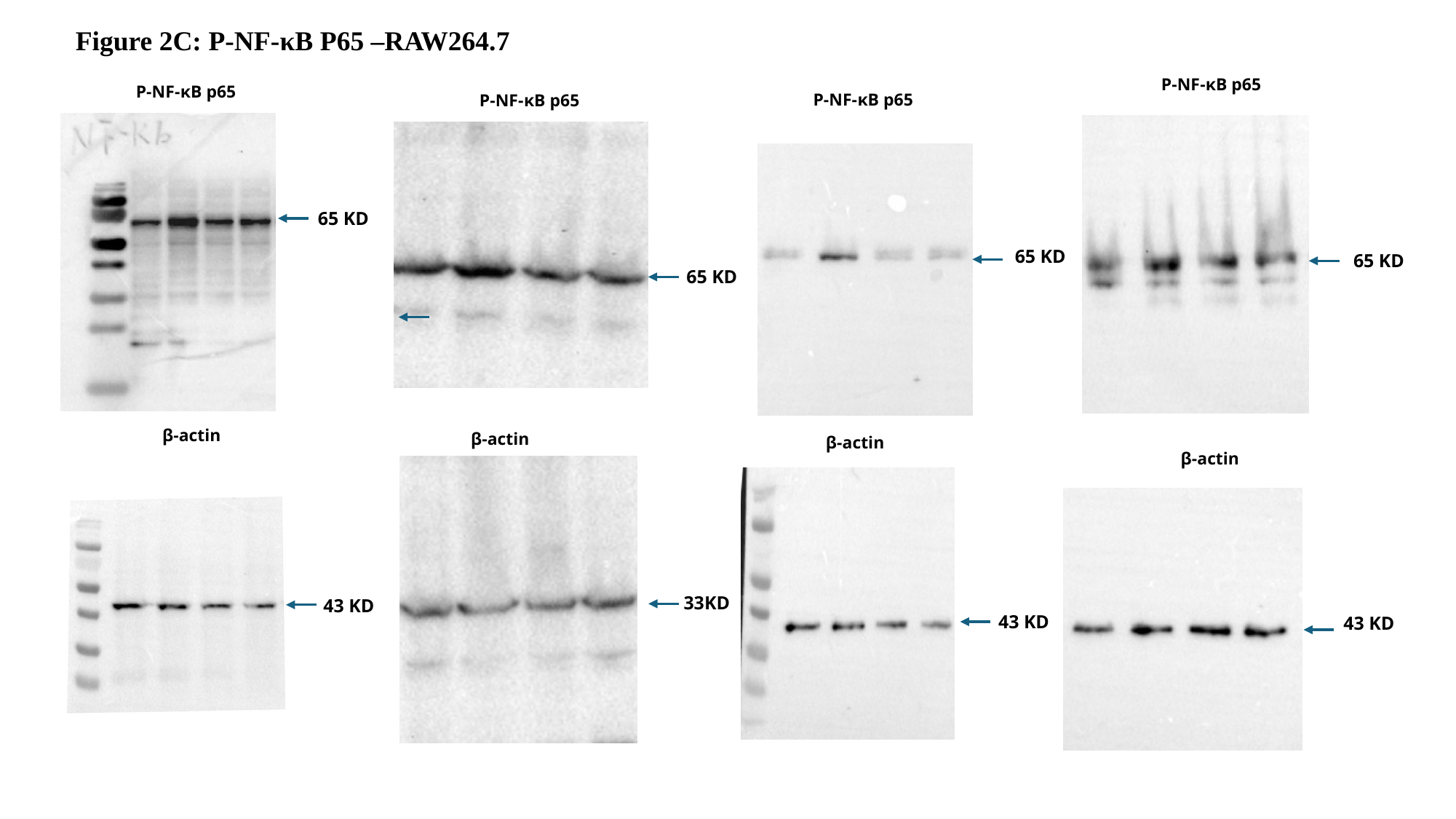

Figure 2C: P-NF-κB P65 –RAW264.7
P-NF-κB p65
P-NF-κB p65
P-NF-κB p65
P-NF-κB p65
65 KD
65 KD
65 KD
65 KD
β-actin
β-actin
β-actin
β-actin
33KD
43 KD
43 KD
43 KD

## Slide 2
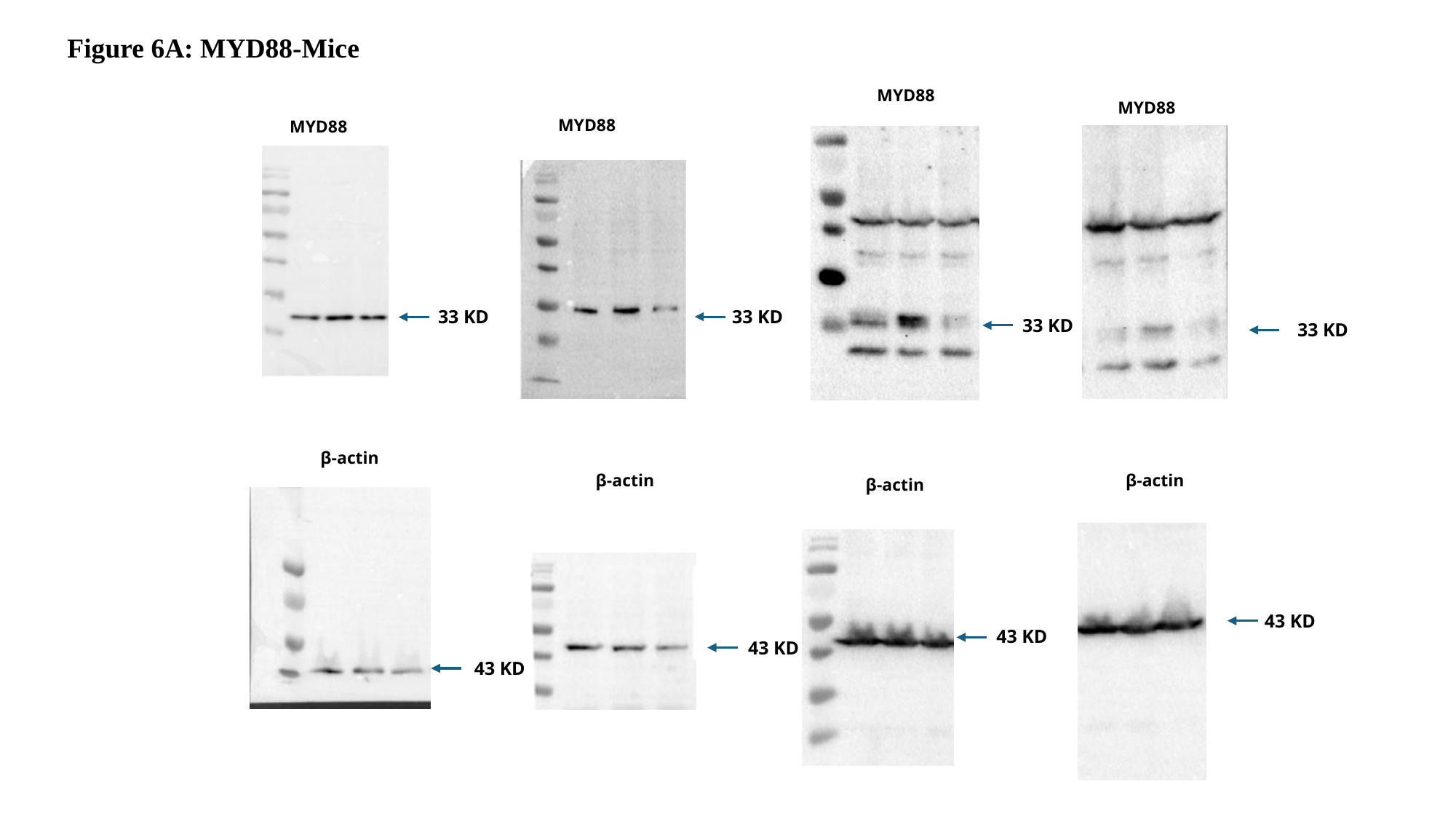

Figure 6A: MYD88-Mice
MYD88
MYD88
MYD88
MYD88
33 KD
33 KD
33 KD
33 KD
β-actin
β-actin
β-actin
β-actin
43 KD
43 KD
43 KD
43 KD

## Slide 3
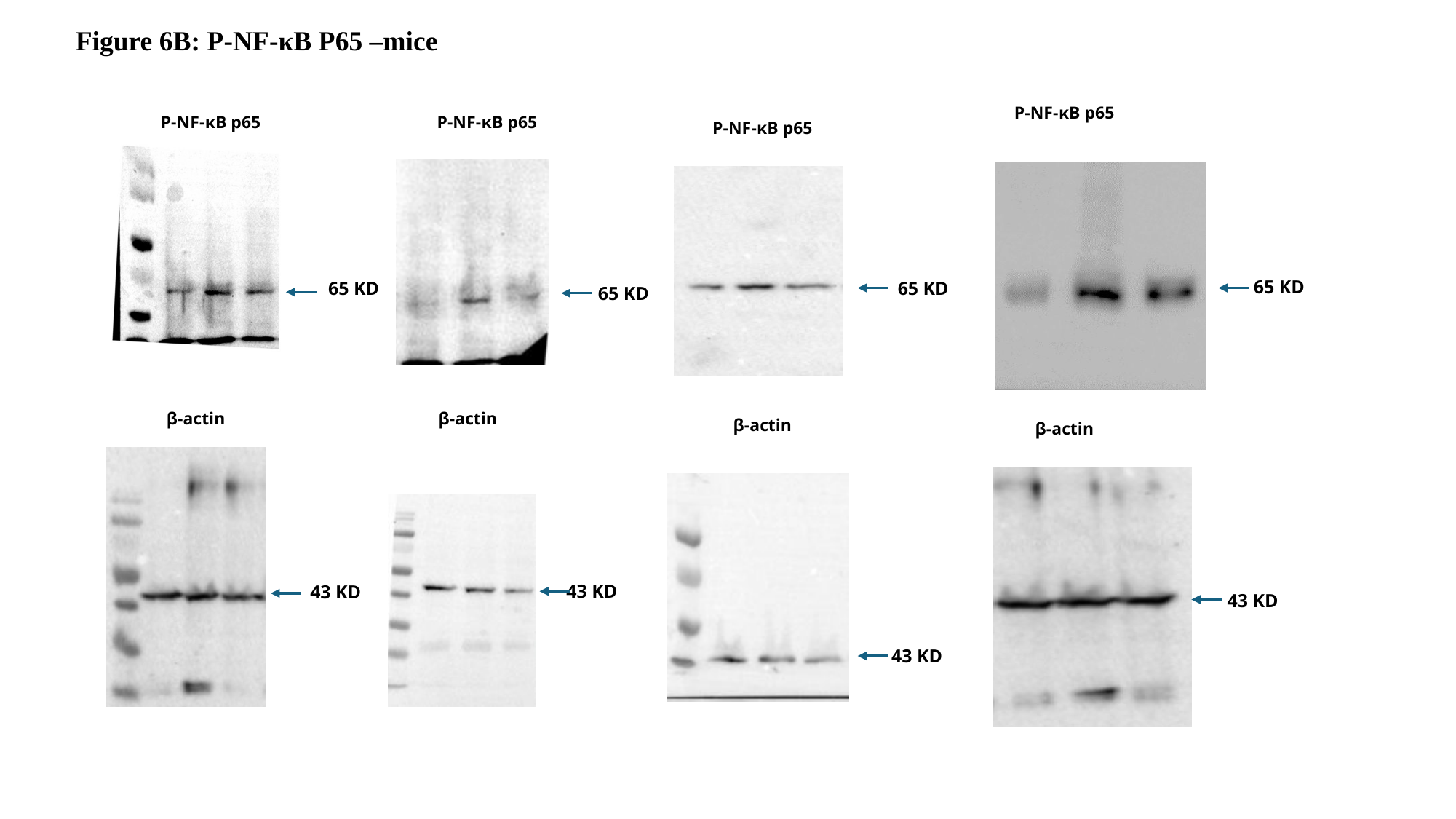

Figure 6B: P-NF-κB P65 –mice
P-NF-κB p65
P-NF-κB p65
P-NF-κB p65
P-NF-κB p65
65 KD
65 KD
65 KD
65 KD
β-actin
β-actin
β-actin
β-actin
43 KD
43 KD
43 KD
43 KD

## Slide 4
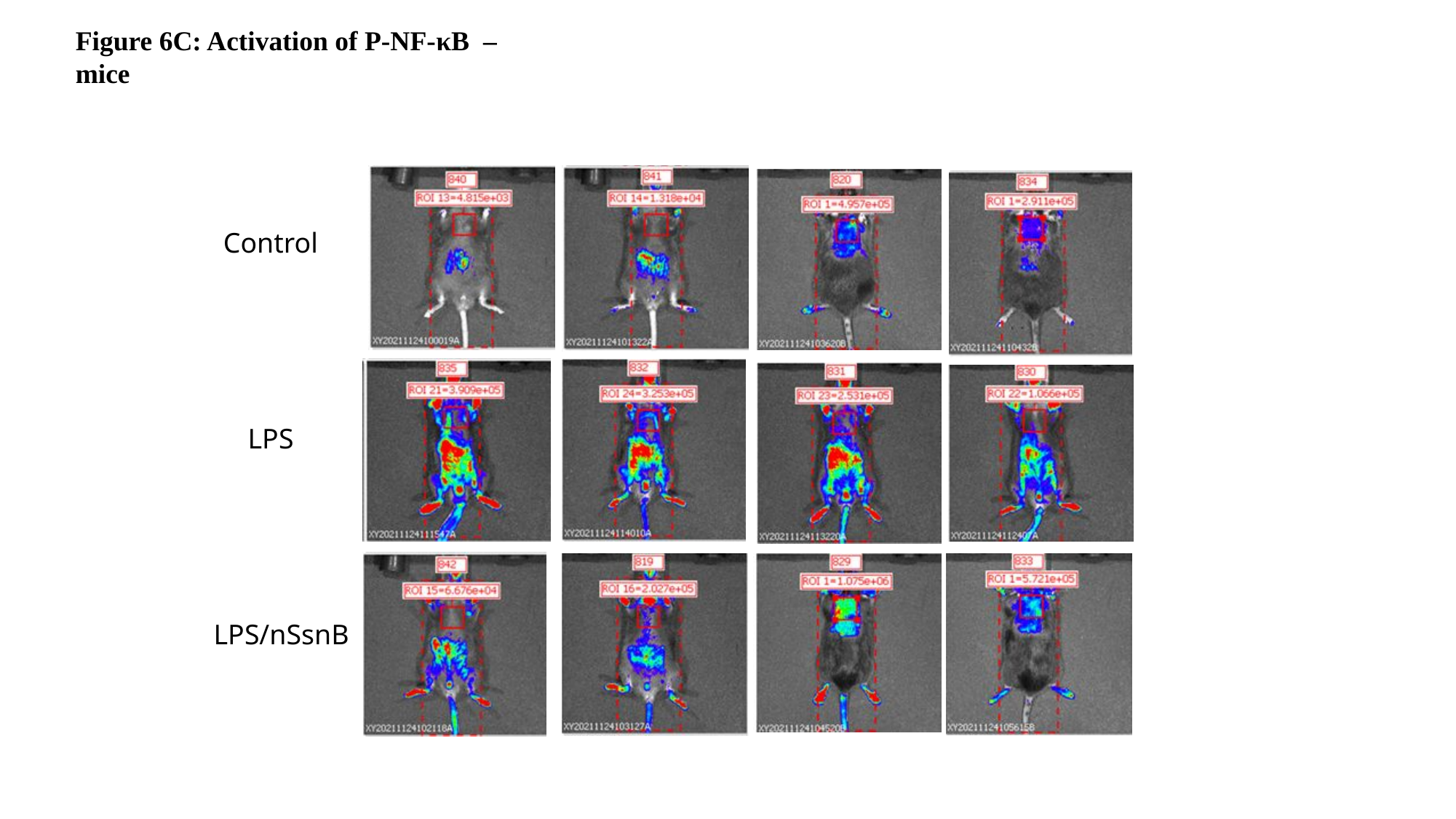

Figure 6C: Activation of P-NF-κB –mice
Control
LPS
LPS/nSsnB
